# Transcriptomic analysis of *Procambarus clarkii* affected by “Black May” disease

**DOI:** 10.1038/s41598-020-78191-8

**Published:** 2020-12-04

**Authors:** Guoqing Shen, Xiao Zhang, Jie Gong, Yang Wang, Pengdan Huang, Yan Shui, Zenghong Xu, Huaishun Shen

**Affiliations:** 1 Wuxi Fisheries College, Nanjing Agricultural University, Nanjing, 210095 China; 2 Key Laboratory of Freshwater Fisheries and Germplasm Resources Utilization, Ministry of Agriculture, Freshwater Fisheries Research Center, Chinese Academy of Fishery Sciences, No. 9 Shanshui East Road, Wuxi, 214081 Jiangsu China

**Keywords:** Transcriptomics, Zoology, Marine biology

## Abstract

Each year from April to May, high mortality rates are reported in red swamp crayfish (*Procambarus clarkii*) cultured in Jiangsu and other regions, in China, and this phenomenon has come to be known as “Black May” disease (BMD). Therefore, in order to investigate the possible causes of this disease, this study gathered BMD-affected *P. clarkii* samples and performed transcriptome analysis on hepatopancreas, gill, and muscle tissues. A total of 19,995,164, 149,212,804, and 222,053,848 clean reads were respectively obtained from the gills, muscle, and hepatopancreas of BMD-affected *P. clarkii*, and 114,024 unigenes were identified. The number of differentially expressed genes (DEGs) in gill, muscle, and hepatopancreas was 1703, 964, and 476, respectively. GO and KEGG enrichment analyses of the DEGs were then conducted. Based on KEGG pathway enrichment analysis, the most significantly differentially expressed pathways were mainly those involved with metabolism, human disease, and cellular processes. Further analysis of the significantly DEGs revealed that they were mainly related to the mitochondrial-mediated apoptosis pathway and that the expression of these DEGs was mostly down-regulated. Moreover, the expression of genes related to immune and metabolism-related pathways was also significantly down-regulated, and these significantly-inhibited pathways were the likely causes of *P. clarkii* death. Therefore, our results provide a basis for the identification of BMD causes.

## Introduction

The red swamp crayfish (*Procambarus clarkii*) is a cambarid crustacean native to the central and southern United States and northeastern Mexico. This species has been successfully introduced into many countries due to its pleasant taste and significant economic value. Moreover, it is also considered the most widespread invasive crayfish species worldwide. *P. clarkii* can be found in freshwater habitats in all continents except Australia and Antarctica^1,2^. This species was introduced to China from Japan in the 1930s and is now widely distributed throughout China's major freshwater bodies and has become an economically-relevant aquatic species in China^3,4^. However, with the continuous increase of farmland areas, a higher frequency of *P. clarkii* diseases is now threatening the development of the aquaculture industry^5,6^.

*P. clarkii* is generally known to be affected by bacterial and viral diseases^1,6,7^. In China, the culture of *P. clarkii* is usually carried out in large-scale outdoor ponds. However, the complexity of these outdoor environments leads to bacterial infection risks. The main pathogens of *P. clarkii* are *Spiroplasma*, *Vibrio*, *Aeromonas hydrophilia*, and *Pseudomonas*, among others. Bacterial infections often cause gill ulcers in *P. clarkii,* which can ultimately lead to death if the conditions are unfavorable^8^. Most of the current research on *P. clarkii* viral diseases largely focuses on the white spot syndrome virus (WSSV). Due to its rapid growth rates, *P. clarkii* is often used as a model organism for WSSV research^2^. However, from April to May of each year since 2008, a large number of cultured *P. clarkii* deaths have been reported in Jiangsu and other regions, in China. This phenomenon has come to be known as “Black May” disease (BMD). The diseased *P. clarkii* appear to reduce food intake, and exhibit a marked redness in their carapace, their carapace is easily peeled off, and their hepatopancreas jejunum appears white. Once a pond becomes affected, a nearly 90% mortality is typically reported. Few studies have addressed BMD in *P. clarkii* either domestically or abroad, and therefore the causes of this disease in *P. clarkii* remain controversial. It is generally believed that BMD in *P. clarkii* is caused by large fluctuations in temperature and water temperature in May. WSSV and some *Vibrio* species reproduce in large numbers and cause proliferative diseases in *P. clarkii*. BMD is also believed to be caused by rapid increases in water temperature, low dissolved oxygen in water bodies, or an accelerated nitrogen cycle due to temperature rise, leading to the accumulation of ammonia nitrogen and nitrite. Most views tend to attribute BMD to the accumulation of environmental stressors^9^. However, there is no firm evidence to support this view. Currently, few studies have addressed the causes of BMD and most studies have primarily focused on the detection of WSSV.

As a highly effective technique for the analysis of gene expression, high-throughput RNA-sequencing (RNA-seq) has been used to identify differentially expressed genes (DEGs), and discovery of novel transcripts^10^. In recent years, the RNA-seq approach has been applied widely to study diseases and immune mechanisms in crustaceans^11–15^. Transcriptome analysis can exhibit the full information about all RNA molecules transcribed by the genome under a certain set of physiological or pathological conditions, especially disease conditions, and can be used to improve understanding of the genes underlying host response to disease^16^.

Therefore, we analyzed the transcriptome of *P. clarkii* affected by BMD through Illumina NovaSeq and bioinformatic analysis and identified DEGs. Our study will help to understand the high lethality pathogenic mechanism of *P. clarkii* affected by BMD, and provide clues regarding the understand the etiology of BMD, which will provide a reference for further research.

## Results

### Histological sections

**Figure 1:**
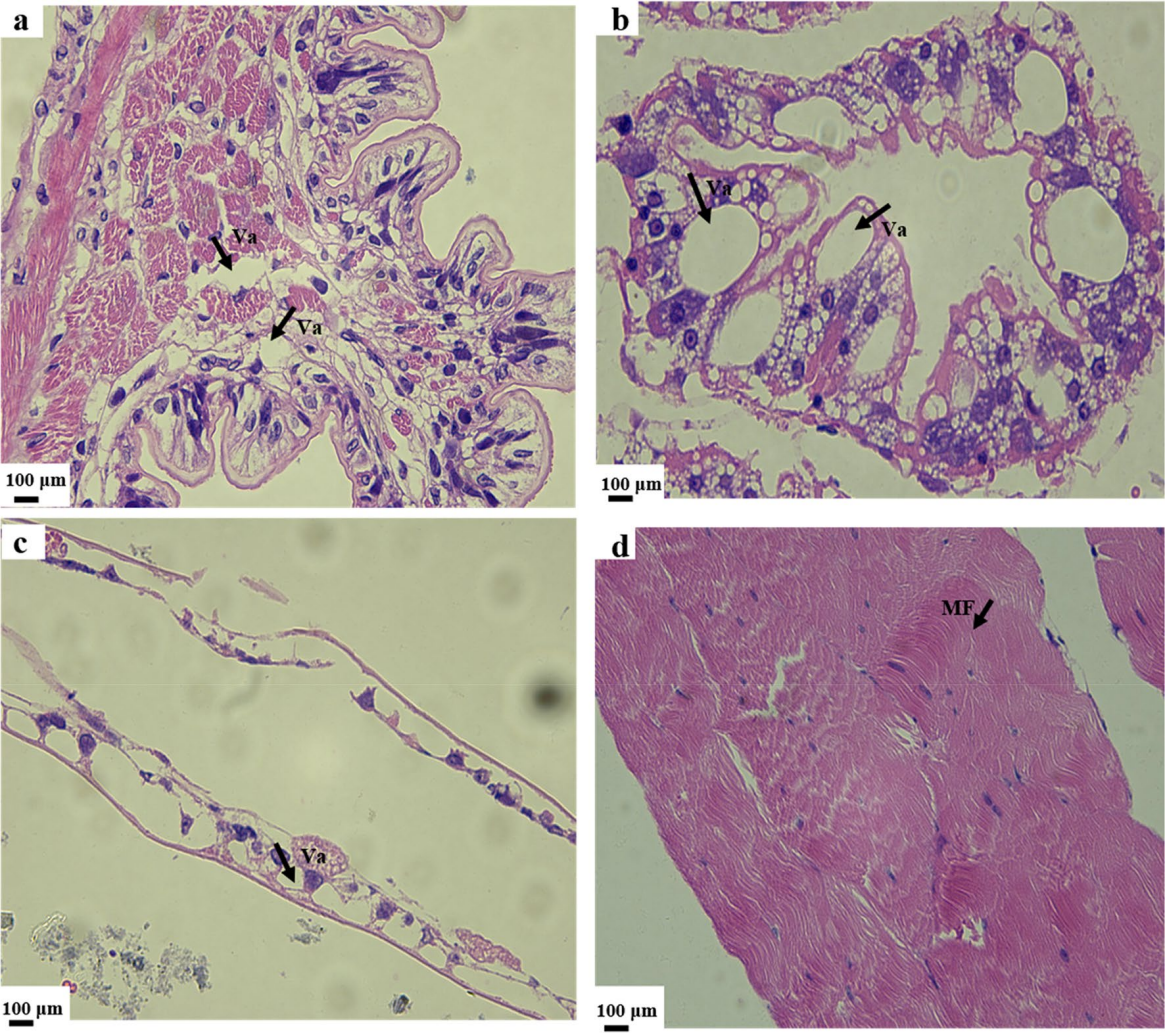
Histological observations of the **(a)** intestine, **(b)** hepatopancreas, **(c)** gills, and **(d)** muscle of dying *P. clarkii* affected by BMD. *Va* vacuole, *MF* muscle fiber.

Dying *P. clarkii* affected by BMD exhibited severe pathological intestinal changes. Specifically, the intestine tissue was loose and some cells presented vacuoles (Fig. 1a). Upon examining the hepatopancreas, we observed that the structure of the hepatopancreas of BMD-affected *P. clarkii* was loose and necrotic. The structure of hepatic tubules disappeared, cells presented edema and were deformed, and a large number of vacuoles were observed (Fig. 1b). Moreover, the gill leaf structure of BMD-affected organisms was severely damaged, the number of cells decreased, the gill leaf exhibited severe vacuolation, and the internal structure of the gill leaf was largely disrupted (Fig. 1c). In muscle tissues, muscle fiber breakage and dissolution was observed (Fig. 1d).

### Sequencing and assembly

Of the 24 samples submitted for sequencing, two samples did not meet quality assurance requirements and were thus discarded. Therefore a total of 22 samples from the six experimental groups were analyzed, meaning that each group contained at least three biological replicates. Approximately 198,595,164, 149,212,804, and 222,053,848 raw reads were obtained from the gill, muscle, and hepatopancreas tissues of diseased shrimp, and 195,105,528, 146,109,594, and 218,446,772 clean reads were obtained after filtering. Furthermore, approximately 141,036,364, 207,578,550, and 204,280,888 raw reads were obtained from gill, muscle, and hepatopancreas tissues of healthy shrimp in the control group, which were then filtered to generate 138,567,790, 204,809,264, and 201,014,454 clean reads, respectively. The Q30 values of the control and the experimental groups were all above 92.11%, and the G + C content was between 42.82% and 49.42%. The detailed sequencing results are summarized in Table 1.

**Table 1:** Transcriptome sequencing summary statistics.

Group	Total raw reads	Total clean reads	Q20 (%)	Q30 (%)	GC (%)
Gill-vir	198,595,164	195,105,528	96.96	92.11	42.82
Hep-vir	222,053,848	218,446,772	97.61	93.49	47.52
Mus-vir	149,212,804	146,109,594	97.22	92.66	48.01
Gill-crt	141,036,364	138,567,790	97.04	92.31	44.02
Hep-crt	204,280,888	201,014,454	97.51	93.25	47.59
Mus-crt	207,578,550	204,809,264	97.60	93.44	49.42

The obtained clean reads were assembled de novo using the Trinity software. A total of 199,094 transcripts were generated, with an average length of 1363 bp, N50 of 2689 bp, a maximum sequence length of 32,536 bp, and a minimum of 301 bp. Moreover, 114,024 unigenes were identified, with an average length of 1029 bp, N50 of 1602 bp, a maximum sequence length of 32,536 bp, and a minimum of 301 bp. Transcript and unigene sizes are illustrated in Supplementary Figure S1.

### Annotation and classification of gene functions

In order to obtain comprehensive data on gene functions, gene function annotations were obtained from seven major databases, including NR, NT, PFAM, KOG/COG, SwissProt, KEGG, and GO. As shown in Supplementary Figure S2, 52,884 unigenes (46.37%) were annotated into the NR database, 36,078 unigenes (31.64%) were annotated into the NT database, 26,491 unigenes (23.23%) were annotated into the KO database, 45,768 unigenes (40.13%) were annotated into the SwissProt database, 47,458 unigenes (41.62%) were annotated into PFAM, 47,458 unigenes (41.62%) were annotated into GO, and 25,607 unigenes (22.45%) are annotated into the KOG database. Among the annotated databases, the NR database was the most annotated.

Analyses of the genome distribution of BLASTX species in the NR database of 52,884 unigenes indicated that, as shown in Supplementary Figure S3, the match rate with *Hyalella Azteca* was the highest (10%), followed by that of *Branchoostoma belcheri* (6%), *Tetrahymena thermophila* (5.6%), *Nephila clavipes* (5.3%), and *Pseudocohnilembus persalinus* (2.95%). These low unigene annotation proportions were largely attributed to a lack of information on the genes or proteins of aquatic crustaceans in the database ^17^.

**Figure 2:**
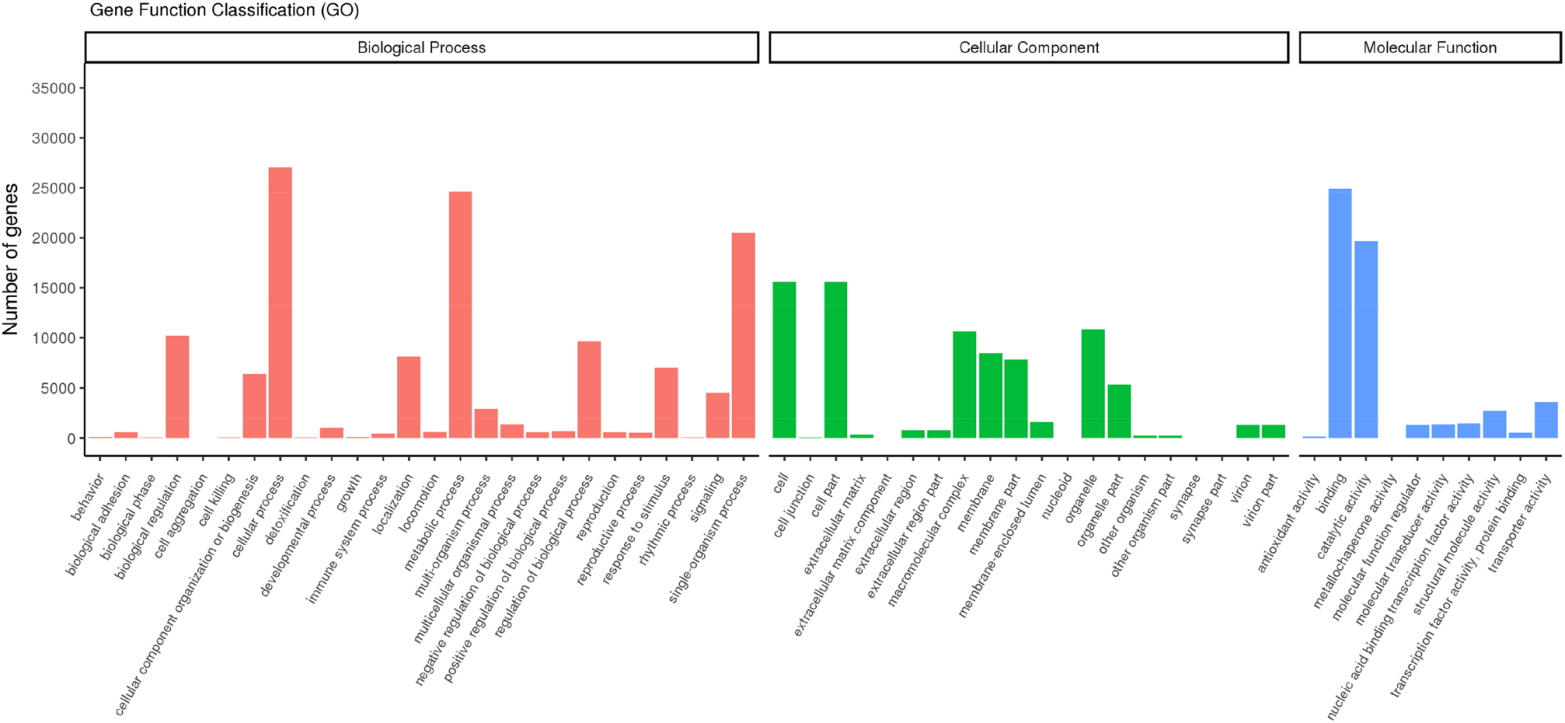
Transcriptome assembly GO terms and annotations. The results are summarized into three ontology categories (cellular component, biological function, and molecular function), and the y-axis indicates the total number of genes in each category.

Based on our GO database sequence homology analysis, a total of 47,458 unigenes were divided into three main GO categories, namely "biological process" (BP), "cellular component" (CC), and "molecular function" (MF). These three main GO categories divide unigenes into 56 sub-categories. Among them, 26 BP sub-categories were obtained. Further, within these BP sub-categories, most unigenes were involved in cellular and metabolic processes, followed by single-organism processes. A total of 20 CC sub-categories were identified and were found to be mainly associated with cell part. Finally, 10 MF sub-categories were identified, and were mainly associated with binding and catalytic activity (Fig. 2).

**Figure 3:**
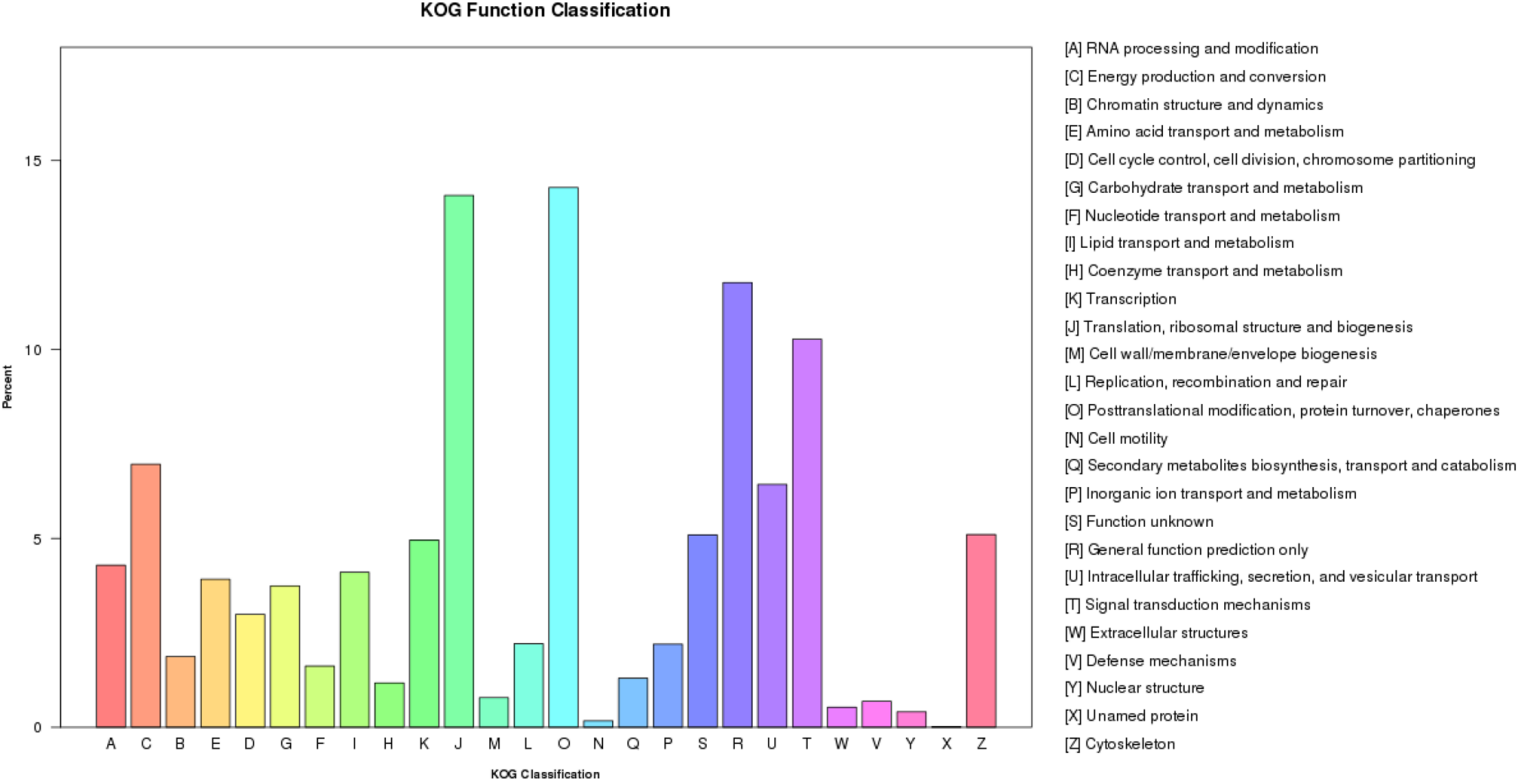
KOG function sequence classification. The x-axis represents the names of 26 groups. The y-axis corresponds to the number of genes in each group.

The largest category in the KOG functional classification was "post-translational modifications, protein turnover, chaperones" (14.28%). The second largest was "translation, ribosomal structure and biogenesis" (14.07%). Moreover, a relatively low proportion of the classifications were associated with general function prediction (11.7%), signal transduction mechanisms (10.26%), and energy production and conversion (6.96%) (Fig. 3).

### Identification of differentially expressed genes (DEGs)

**Figure 4:**
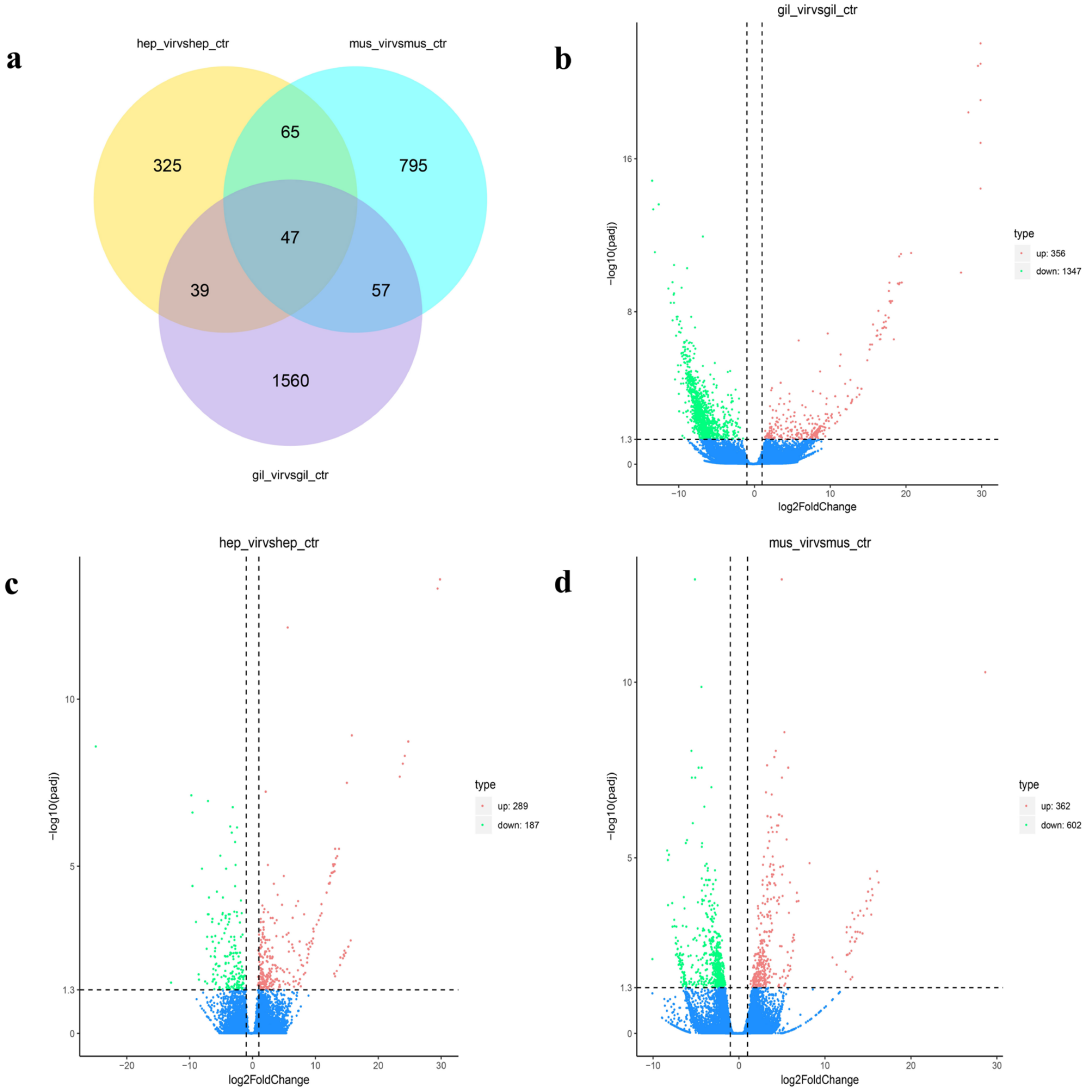
**(a)** Venn diagram of the differentially expressed genes in hepatopancreas, muscle, and gill tissues. Volcano plots of DEG distribution trends between **(b)** Gill-vir and Gill-crt, **(c)** Hep-vir and Hep-crt, and **(d)** Mus-vir and Mus-crt. The x-axis indicates the fold change between two sets of samples, and the y-axis shows the significance of the DEGs. Red (up-regulation) and green (down-regulation) dots represent significantly different expressions (P-value < 0.05), whereas blue dots indicate non-significant differences.

**Figure 5:**
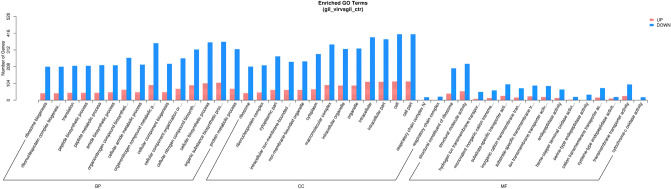
GO terms and annotation of DEGs. The results were summarized into three ontologies (cellular component, biological function, and molecular function). The y-axis indicates the total number of genes in each category. Red (up-regulation) and blue (down-regulation).

As illustrated in Fig. 5, the number of DEGs (i.e., relative to the tissues of healthy *P. clarkii*) in the gills, muscle, and hepatopancreas of *P. clarkii* infected with BMD was 1,703, 964, and 476, respectively (Fig. 4a). Notably, the gills exhibited the highest number of DEGs, among them, there are 356 up-regulated genes and 1,347 down-regulated genes. (Fig. 4b). The number of DEGs in the hepatopancreas was the lowest (only 476), among them, there are 289 up-regulated genes and 187 down-regulated genes (Fig. 4c). The muscle tissue exhibited a total of 964 DEGs, of which 362 were up-regulated and 602 were down-regulated (Fig. 4d).

### GO and KEGG enrichment analysis of DEGs

A total of 1703 DEGs in gills were classified into three categories in GO annotations, namely biological process (BP), cellular component (CC), and molecular function (MF). In the biological process category, DEGs were divided into 20 sub-categories. Among these sub-categories, biosynthetic process (GO: 0009058, 41.19%), organic substance biosynthetic process (GO: 1901576, 41.01%), and cellular biosynthetic process (GO: 0044249, 40.4%) were the most highly represented. In the cell component category, DEGs were divided into 20 sub-categories. In these sub-categories, DEGs were mainly concentrated in the cell part (GO: 0044464, 45.81%), cell (GO: 0005623, 45.81%), and intracellular (GO: 0005622, 44.07%) categories. Moreover, among the 20 sub-categories of molecular functions, DEGs were mainly associated with structural molecule activity (GO: 0005198, 24.52%) and structural constituent of ribosome (GO: 0003735, 20.86%) (Fig. 5).

**Figure 6:**
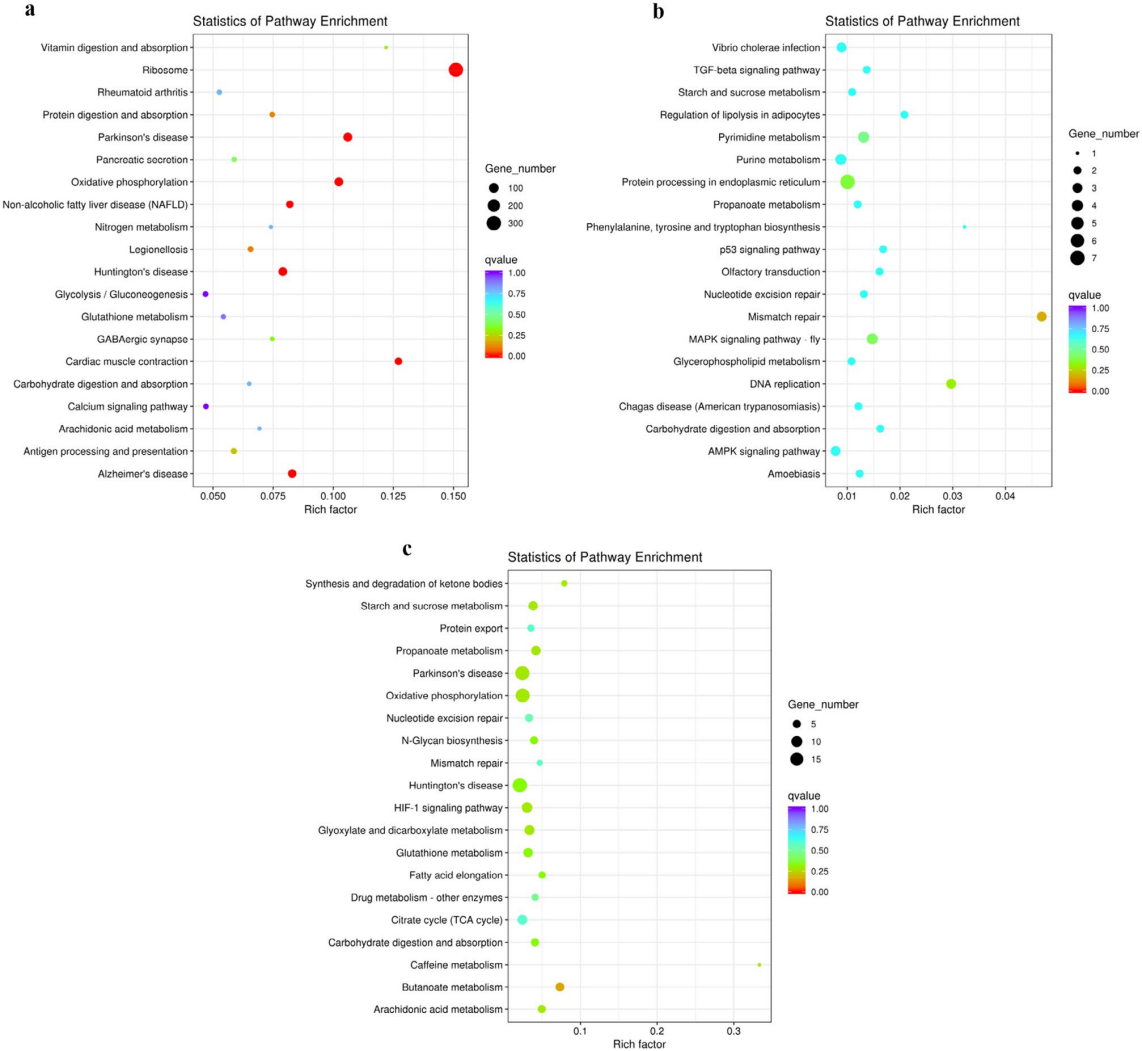
KEGG pathway analysis between **(a)** Gill-vir and Gill-ctr, **(b)** Hep-vir and Hep-ctr, and **(c)** Mus-vir and Mus-ctr.

KEGG enrichment analysis was conducted on the DEGs identified in different tissues. Gills are the main respiratory-related organs of aquatic crustaceans, and therefore play an important role in external osmotic pressure regulation, ion transport, and immune response^18–20^. KEGG enrichment results (Fig. 6a) indicated that human disease pathways were significantly enriched, and the DEGs were mainly enriched in the ribosome, Parkinson's disease, oxidative phosphorylation, cardiac muscle contraction, antigen processing and presentation, carbohydrate digestion and absorption, N metabolism, and glycolysis categories. Ribosome-related gene pathways represented the largest group in our gill KEGG analysis. Ribosomes are the main organelles responsible for protein synthesis in cells, and therefore play an important role in regulating cell physiology and gene functions^21–23^. Oxidative phosphorylation is an important pathway for cell metabolism, and it is also one of the main mechanisms for organisms to obtain energy. The expression levels of most of the genes within these pathways were decreased.

The hepatopancreas is an important immune organ in crustaceans, it plays an important role in regulating the metabolic processes and immune responses of living organisms^3,24–26^. Among the KEGG enrichment results (Fig. 6b), the most significantly enriched pathways were mismatch repair, DNA replication, and protein processing in the endoplasmic reticulum, followed by metabolism and immune-related pathways, pyrimidine metabolism, purine metabolism, regulation of lipolysis in adipocytes, glycerophospholipid metabolism, and propionate metabolism. Among the immune-related pathways, the P53 signaling pathway, MAPK signaling pathway-fly, TGF-beta signaling pathway, and AMPK signaling pathway were significantly enriched.

The most significantly enriched pathways in muscle tissue were mainly associated with metabolism (Fig. 6c), including starch and sucrose metabolism, pyrimidine metabolism, purine metabolism, propanoate metabolism, glycerophospholipid metabolism, carbohydrate digestion and absorption.

Among the top 30 DEGs, many pathways were related to the innate immunity of crustaceans, cell growth and death-related apoptosis, phagosomes, lysosomes, endocytosis in transport and catabolism, tight junctions, adhesions in autophagy, animals, cell communities, eukaryotes, regulation of actin cytoskeleton related to cell movement, and PI3K-Akt signaling pathway, MAPK signaling pathway, and the cGMP-PKG signal pathway.

### DEGs and pathways

**Figure 7:**
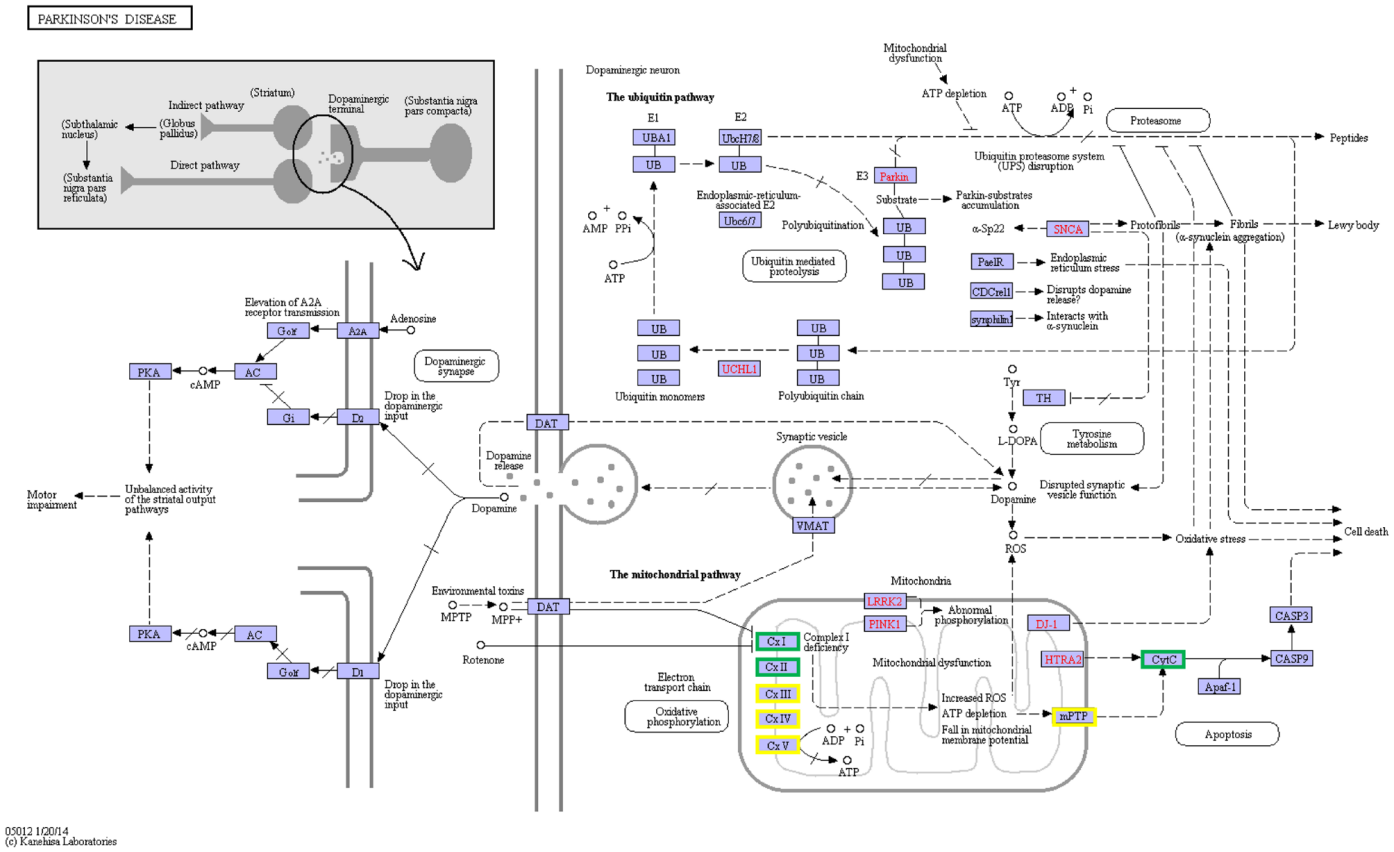
Parkinson's disease pathway. KO node borders containing down-regulated genes are marked in green, and borders containing up-down genes are marked in yellow. (https://www.kegg.jp/kegg/kegg1.html).

Upon conducting DEG and KEGG enrichment analyses, pathways associated with Parkinson’s disease, cardiac muscle contraction, Alzheimer's disease, Huntington’s disease, and Non-alcoholic fatty liver disease (NAFLD) were found to be significantly affected, as well as mitochondrial-mediated apoptotic pathways (Table 2). Mitochondria generally control apoptosis through three main mechanisms: (1) disrupting the electron transfer chain and thus halting ATP synthesis; (2) triggering the release of proteins that activate apoptosis, and (3) changing the cell’s redox potential^27^. The electron transport chain works through four protein complexes distributed on the inner membrane of the mitochondria. Figure 7 demonstrates that the expression of the protein complexes in the electron transport chain, including respiratory chain complex I, complex II, complex III, complex IV, and complex V, were significantly down-regulated (Table 3)^28^. This indicates that the electron transfer chain was adversely affected. Mitochondrial respiratory chain complex I, also known as NADH dehydrogenase, is the first protein complex in the electron transport chain. In the diseased gills of *P. clarkii*, complex I with ND1, ND3, ND4, and ND5 related genes decreased. Moreover, the expression of NDUFAB1-, NDUFB9-, NDUFB10-, and NDUFB11-related genes decreased significantly. Mitochondrial respiratory chain complex II, also known as "succinate dehydrogenase," is the second component of the electron transport chain, and is the only enzyme that is involved in both the tricarboxylic acid cycle and oxidative phosphorylation. Notably, SDHD (i.e., succinate dehydrogenase complex subunit D) expression was found to be significantly down-regulated. Furthermore, mitochondrial respiratory chain complex III, also known as “electron transfer-flavin protein dehydrogenase,” is the third component of the electron transfer chain. It is an enzyme that accepts electrons from the mitochondrial matrix to transfer flavin proteins and uses these electrons to reduce ubiquinone. Among the CYTB-encoding genes in the gills of the diseased *P. clarkii*, the expression of 4 genes decreased significantly, the expression of 2 genes increased. Complex IV, also called cytochrome C oxidase, is the last protein complex in the electron transfer chain. This enzyme carries the final reaction of the electron transfer chain and transfers electrons to oxygen when pumping protons across the membrane. At this point, oxygen is reduced to water as the final electron acceptor. The genes encoding COX2, COX4, COX5A, COX5B, and COX6B were all significantly down-regulated in the gills of the diseased *P. clarkii*. The expression of five genes encoding the COX3 gene decreased, whereas that of two related genes increased. Moreover, there was a significant decrease in ATPeF and ATPeV-related genes in complex V.

**Table 2:** Mitochondrial-mediated apoptosis-related KEGG pathways.

Pathway	DEGs	Background number	P-value	Pathway ID
Parkinson's disease	79	745	7.28E−18	ko05012
Oxidative phosphorylation	75	733	2.88E−16	ko00190
Alzheimer's disease	66	796	9.43E−11	ko05010
Huntington's disease	72	911	9.82E−11	ko05016
Non-alcoholic fatty liver disease (NAFLD)	44	537	1.62E−07	ko04932

**Table 3:** Differentially expressed genes associated with mitochondrial-mediated apoptosis pathways.

Gene ID	Gene name	Description	log2FC	Ko ID
Cluster-26245.15953	ND1	NADH-ubiquinone oxidoreductase chain 1	− 8.1211	K03878
Cluster-47055.0	ND3	NADH-ubiquinone oxidoreductase chain 3	− 6.2133	K03880
Cluster-39778.0	ND4	NADH-ubiquinone oxidoreductase chain 4	− 7.9973	K03881
Cluster-26245.15635	NDUFAB1	NADH dehydrogenase (ubiquinone) 1 alpha/beta subcomplex 1	− 6.2684	K03955
Cluster-26245.32897	NDUFB9	NADH dehydrogenase (ubiquinone) 1 beta subcomplex subunit 9	− 5.9799	K03965
Cluster-55302.0	NDUFB11	NADH dehydrogenase (ubiquinone) 1 beta subcomplex subunit 11	− 7.0493	K11351
Cluster-34059.0	NDUFB10	NADH dehydrogenase (ubiquinone) 1 beta subcomplex subunit 10	− 6.323	K03966
Cluster-44106.0	SDHD, SDH4	Succinate dehydrogenase (ubiquinone) membrane anchor subunit	− 6.4843	K00237
Cluster-43914.0	CYC	Cytochrome c	− 6.7258	K08738
Cluster-26245.30195	CYTB	Ubiquinol-cytochrome c reductase cytochrome b subunit	− 9.1728	K00412
Cluster-55533.0	COX2	Cytochrome c oxidase subunit 2	− 7.307	K02261
Cluster-42690.0	COX5B	Cytochrome c oxidase subunit 5b	− 6.6969	K02265
Cluster-44154.0	COX4	Cytochrome c oxidase subunit 4	− 6.4834	K02263
Cluster-40220.0	COX5A	Cytochrome c oxidase subunit 5a	− 6.1701	K02264
Cluster-26245.34944	COX6B	Cytochrome c oxidase subunit 6b	− 7.843	K02267
Cluster-26245.15908	COX3	Cytochrome c oxidase subunit 3	− 5.8606	K02262
Cluster-55525.0	VDAC1	Voltage-dependent anion channel protein 1	− 7.1415	K05862

Mitochondrial membrane permeability transition pores (mPTP) are non-specific channels formed by various mitochondrial proteins located between the inner and outer membranes of the mitochondria, which play an important role in energy metabolism and oxidative stress response^29,30^. Based on our transcriptome data, the expression of the VDAC1 gene (i.e., which encodes an mPTP-related protein) was significantly down-regulated. Cytochrome C plays an important role in controlling apoptosis, and therefore its absence or dysfunction leads to abnormal function of the mitochondrial respiratory chain, causing ATP deficiency to cause cell death. In our results, the expression of cytochrome C decreased.

## Discussion

Due to the high lethality of BMD and the complexity of the weather from April to May, the different tissues of *P. clarkii* are adversely affected by pathogens and environmental stressors, as well as the resulting gene expression changes. Gills are the main respiratory-related organs of aquatic crustaceans and thus play an important role in the regulation of external osmotic pressure, ion transport, and immune response^18–20^. Based on our transcriptome data, the gills of diseased *P. clarkii* presented the highest number of differentially expressed genes compared to the two other tissues examined herein (hepatopancreas and muscle). Moreover, gill tissues exhibited more down-regulated than up-regulated genes. In other words, gene expression was largely inhibited, especially in genes associated with respiratory metabolism and related pathways such as oxidative phosphorylation.

The hepatopancreas is an important immune organ in crustaceans and plays an important role in regulating the metabolic processes and immune responses of living organisms. Based on our transcriptome data, the DEGs in the hepatopancreas were mainly related to immune and metabolic pathways, whereas DEGs in the muscle were mainly associated with metabolic pathways^3,24–26^.

Invertebrates such as *P. clarkii* are generally thought to only have an innate immune system and lack an adaptive immune system. Therefore, when infected by pathogens, *P. clarkii* can only largely rely on its innate immune system^31–33^. The innate immune system of invertebrates relies on pattern recognition receptors (PRRs) and pathogen-associated molecular patterns (PAMPs). This limited immune system makes crustaceans particularly vulnerable to viruses and bacteria. The primary defense mechanisms of crustaceans against bacterial and viral infections are also diverse^6,34^. In BMD-affected *P. clarkii*, dysregulated genes were found to be significantly enriched in classifications associated with apoptosis, autophagy, phagosomes, lysosomes, endocytosis, P53 signaling pathway, PI3K-Akt signaling pathway, and MAPK signaling pathway. Moreover, significant changes were observed in pathways related to innate immune system. Apoptosis is generally defined as programmed cell death, and can therefore serve as a mechanism to remove damaged or redundant cells derived from unfavorable conditions via the caspase signaling pathway. It is an important immune process for crustaceans to resist pathogen infection^35–39^. When a host cell is infected with a virus, it induces an apoptotic response to eliminate the infected cells, which seriously hinders the replication of the virus. This process is often related to mitochondrial-mediated apoptosis pathway, and mitochondrial-mediated apoptosis often induces the release of pro-apoptotic factors including cytochrome C and other mitochondrial factors through changes in mitochondrial outer membrane permeability (MOMP)^40–42^. Therefore, viral infections prolong replication times in the host cell by inhibiting the occurrence of the apoptotic reaction^39,43,44^. In aquatic animals, many studies have revealed that WSSV infection can lead to the inhibition of host cell apoptosis through multiple pathways, thereby controlling the replication of the virus in the host^30,45^. Interestingly, in our results, the expression of cytochrome C in *P. clarkii* affected by BMD was suppressed. Cytochrome C absence or dysfunction leads to abnormal function of the mitochondrial respiratory chain, causing ATP deficiency to cause cell death. The P53 pathway is the main apoptosis-related signaling pathway. Notably, p53 has been shown to regulate the activity of apoptosis in favor of WSSV infection in WSSV-infected *Marsupenaeus japonicas*^46^. Moreover, lysosomes play an important role in humoral immunity (i.e., a type of innate immunity) by secreting a variety of soluble acid hydrolases, thereby causing bacterial cell lysis^6,47^. Particularly, lysozyme expression becomes up-regulated as an innate immune response mechanism in shrimps infected with bacterial pathogens^48,49^. Cathepsins are an important group of proteases in lysosomes. Cathepsin L and Cathepsin B is an important member of the cathepsin family^42^. In our results, the expression of Cathepsin L and Cathepsin B in *P. clarkii* affected by BMD was induced. Phagocytosis is another important innate immunity defense mechanism and plays an important role in the identification and elimination of invading microbial pathogens. Calreticulin (CRT) is an endoplasmic reticulum (ER) luminal resident protein and involved in a variety of biological processes, including growth, reproduction, molting of crustaceans, immune response, apoptosis, and oxidative stress response^50–52^. Interestingly, in our results, the expression of CRT in *P. clarkii* affected by BMD was down-regulation. Down-regulation of CRT expression may be related to immunodepression. In conjunction, lysosomes, autophagy, phagosomes, and endocytosis form the humoral immune system^53^. Yang et al. studied the differences in the gene expression of *P. clarkii* in response to WSSV and *Aeromonas hydrophila* through comparative transcriptomics. Apoptosis-related pathways and other related genes were differentially expressed in bacteria-infected organisms compared with those infected with WSSV, and the main affected pathways were associated with endocytosis, focal adhesions, phagosomes, MAPK signaling pathways, and other immune-related pathways^6^. These results are consistent with the effects on immune-related pathways observed herein in BDM-affected *P. clarkii*. Moreover, apoptotic-related pathways were also significantly enriched. We speculate that viral infections may be related to the onset of “Black May,” and differentially expressed genes were significantly enriched in humoral immune-related pathways. The disease might also be caused by bacteria. However, further studies are required to more accurately determine whether BMD is indeed caused by WSSV, other viruses, or bacteria.

The voltage-dependent anion channel (VDAC) of the outer mitochondrial membrane is a protein that is associated with the mitochondrial membrane permeability transition pore (mPTP). Many studies have confirmed that when the host is infected with a virus, VDAC proteins tend to accumulate during the infection^54–56^. Shrimps infected with WSSV exhibited up-regulated VDAC expression. However, when VDAC was silenced, WSSV-induced mortality and virion copy numbers were reduced, and infection was delayed, indicating that VDAC is necessary for WSSV replication^30,57,58^. Interestingly, VDAC gene expression was significantly down-regulated in our experimental results, which may have been caused by impaired mitochondrial functions in *P. clarkii*.

KEGG enrichment analysis of DEGs identified several significantly affected metabolic-related pathways, including starch and sucrose metabolism, pyrimidine metabolism, purine metabolism, propanoate metabolism, glycerophospholipid metabolism, carbohydrate digestion and absorption. Notably, most of the DEGs that comprise the aforementioned pathways were found to be down-regulated. Nian et al. studied the transcriptome of IHHNV-infected *P. clarkii* and found that the expression of genes within pathways related to carbohydrate absorption and metabolism were down-regulated in the digestive tract of infected organisms^59^. These observations may explain the jejunum abnormalities observed in BMD-affected *P. clarkii*.

## Conclusion

This study sought to identify the molecular mechanisms of BMD onset, which causes enormous losses in the *P. clarkii* breeding industry each year. Currently, few studies have addressed BMD, and most of them focus on the detection of WSSV. A transcriptomics approach was used to study gene expression differences in gill, muscle, and hepatopancreas tissues of *P. clarkii* affected BMD. A total of 199,094 transcripts were obtained, and 1,703, 964, and 476 differentially expressed genes were identified in the gills, muscle, and hepatopancreas of BMD-affected organisms. KEGG and GO enrichment analyses were conducted on the DEGs, which revealed that the expression of metabolic- and immune-related genes was significantly down-regulated. Significantly enriched KEGG pathways were mainly related to mitochondrial-mediated apoptosis. The changes in these pathways may have eventually led to the death of *P. clarkii*. Therefore, our findings provide insights into the molecular mechanisms of BMD onset in *P. clarkii*.

## Materials and methods

### Sampling

The diseased crayfish used in the experiments were collected from a *P. clarkii* farm affected by BMD in Xuyi County, Jiangsu Province, China. The organisms exhibited lethargy, and jejunum abnormalities. The gills, muscles, and hepatopancreas of sick organisms were immediately recovered, flash-frozen in liquid nitrogen, and stored at − 80 °C. Four samples were taken from each experimental group, for a total of 12 samples. Moreover, twelve healthy *P. clarkii* samples were then collected, and their gill, muscle, and hepatopancreas tissues were similarly recovered and stored in liquid nitrogen to be used as a control group. Another subset of approximately 6 mm^3^ gill, muscle, hepatopancreas, and intestine samples was also recovered and fixed with a 4% paraformaldehyde solution at room temperature for two hours, then stored at − 4 °C for later use.

### Preparation of histological sections

Tissue blocks fixed with 4% paraformaldehyde were subjected to tissue dehydration and paraffin embedding. The wax blocks were then cut into tissue sections (5 μm), after which the sections were subjected to hematoxylin–eosin (HE) staining. The prepared samples were then inspected with a microscope.

### Total RNA extraction

Each collected tissue was lysed with TRIzol reagent (Takara, Beijing, China) according to the manufacturer's instructions to extract total RNA and then treated with DNase I to remove genomic DNA. Afterward, 1% agarose gel electrophoresis was conducted to analyze RNA integrity and DNA contamination. RNA purity (OD260/280 and OD260/230 ratios) was then detected with a NanoPhotometer spectrophotometer (IMPLEN, CA, USA), after which a Qubit 2.0 fluorometer (Life Technologies, CA, USA) was used to accurately quantify RNA concentration. Finally, an Agilent 2100 bioanalyzer (Agilent Technologies, CA, USA) was used to accurately detect RNA integrity.

### cDNA library construction and sequencing

The cDNA library used for all downstream analyses was constructed and sequenced by Beijing Novogene Biosciences. Total RNA from each group was enriched with polyA tailed mRNA using Oligo (dT) magnetic beads. The obtained mRNA was randomly interrupted with a divalent cation in an NEB ragmentation buffer. The fragmented mRNA was used as a template, and random oligonucleotides were used as primers. cDNA was synthesized in an M-MuLV reverse transcriptase system. Afterward, RNaseH was used to degrade the RNA strand and DNA polymerase I was used in conjunction with dNTPs as raw materials to synthesize the second strand of cDNA. The purified double-stranded cDNA was repaired at the end, and an A tail sequencing adapter was added. cDNAs of approximately 250–300 bp were screened with AMPure XP beads and amplified by PCR, after which the PCR products were purified again with AMPure XP beads to finally obtain a library. Upon completing the library construction, preliminary library quantification was conducted in the Qubit 2.0 Fluorometer and diluted to 1.5 ng/µl. The Agilent 2100 bioanalyzer was then used to detect the library insert size. After the insert size was estimated, qRT-PCR was performed with an effective library concentration. Accurate quantification (the effective library concentration was > 2 nM) was conducted to ensure the quality of the library. The library preparation was sequenced on an Illumina Hi-Seq sequencer (Beijing Novogene Biosciences Co., Ltd., Beijing, China).

### Sequence assembly and gene function annotation

In order to ensure the quality and reliability of our data analyses, all raw sequencing data were filtered. Reads with adapters were removed, N-containing reads were removed, and reads with a proportion of bases with Qphred ≤ 20 accounting for more than 50% of the entire read length were considered low quality. The filtered clean reads were spliced using the Trinity (v2.5.1) software (Grabherr et al., 2011)^60^. To obtain Trinity stitching fasta sequences, the BUSCO software was used to evaluate the stitching quality of the Trinity.fasta, unigene.fasta, and cluster.fasta sequences obtained from stitching. Upon completing these steps, the obtained unigene annotations were uploaded to seven major databases, including NR (NCBI non-redundant protein sequences), NT (NCBI nucleotide sequences), PFAM (Protein family), Swiss-Prot (A manually annotated and reviewed protein sequence database), KEGG (Kyoto Encyclopedia of Genes and Genomes), and GO (Gene Ontology).

### Differentially expressed gene (DEG) and enrichment analyses

The RSEM software package was used to calculate the bowtie comparison results. Moreover, the number of read counts for each gene and each sample was compared and FPKM-converted to analyze gene expression^61,62^. DESeq2 was then used to assess differential gene expression based on the negative binomial distribution^63^. Genes with an adjusted P-value < 0.05 found by DESeq were assigned as differentially expressed.

### GO and KEGG enrichment analysis

GO (i.e., gene ontology) enrichment analysis allows for the identification of GO function entries that are significantly enriched when provided with a list of DEGs relative to the genomic background, thereby providing a description of the biological functions associated with the DEGs. The GO-seq method was used to perform GO enrichment analysis on the differential genes for each group^64^. The analysis first matches the differentially expressed genes to each term in the Gene Ontology database (http://www.geneontology.org/), calculates the number of genes in each term, and identifies significantly-enriched gene pathways associated with the differentially expressed genes compared to the genome background.

Different genes coordinate the biological functions of living organisms, and significant pathway enrichment can be an indicator of which biochemical metabolic pathways and signal transduction pathways were most affected based on the DEGs. The KEGG (Kyoto Encyclopedia of Genes and Genomes) database is the main public database for gene pathway annotation^65–67^. Here, the Kobas software was used to perform KEGG pathway enrichment analysis on the DEG results for each group^68,69^.

## Supplementary information

Supplementary Figures.

## Data Availability

Raw sequence data associated with this project have been deposited at NCBI with bioproject accession number PRJNA633285 and SRA accession numbers from SRR11802326 to SRR11802347.
