# Supplementary material for: Transcriptomic analysis of Procambarus clarkii affected by “Black May” disease
Source: Sci Rep. 2020 Dec 4;10:21225. doi: 10.1038/s41598-020-78191-8 (PMC7719172; doi:10.1038/s41598-020-78191-8)

**Transcriptomic analysis of** ***Procambarus clarkii* affected by “Black May”** **disease**

Guoqing Shen^1,2^, Xiao Zhang^1,2^, Jie Gong^1,2^, Yang Wang^1,2^, Pengdan Huang^2^, Yan Shui^2^, Zenghong Xu^2^, and Huaishun Shen^1,2, *^

*^1^ Wuxi Fisheries College, Nanjing Agricultural University, Nanjing 210095, China;*

*^2^ Key Laboratory of Freshwater Fisheries and Germplasm Resources Utilization, Ministry of Agriculture, Freshwater Fisheries Research Center, Chinese Academy of Fishery Sciences, Wuxi 214081, China*

^*^Corresponding authors.

E-mail: [shenhuaishun@ffrc.cn](mailto:shenhuaishun@ffrc.cn),

Full postal address: Freshwater Fisheries Research center of Chinese Academy of Fisheries Sciences No. 9 Shanshui East Road, Wuxi, Jiangsu, China, 214081

**Supplementary Figures**

**Supplementary Figure S1.** Length distribution of unigene and transcript sequence lengths. Blue and red bars indicate unigenes and transcripts, respectively.

**Supplementary Figure S2.** Annotation results against the GO, KO, KOG, NR, NT, PFAM, and SwissProt databases. The bars indicate the number of unigenes that were annotated by each of these databases, and the percentage indicates the proportion of annotated unigenes from the total reads.

**Supplementary Figure S3.** NR unigene homologous species distributions.

**Supplementary Figure S1.**


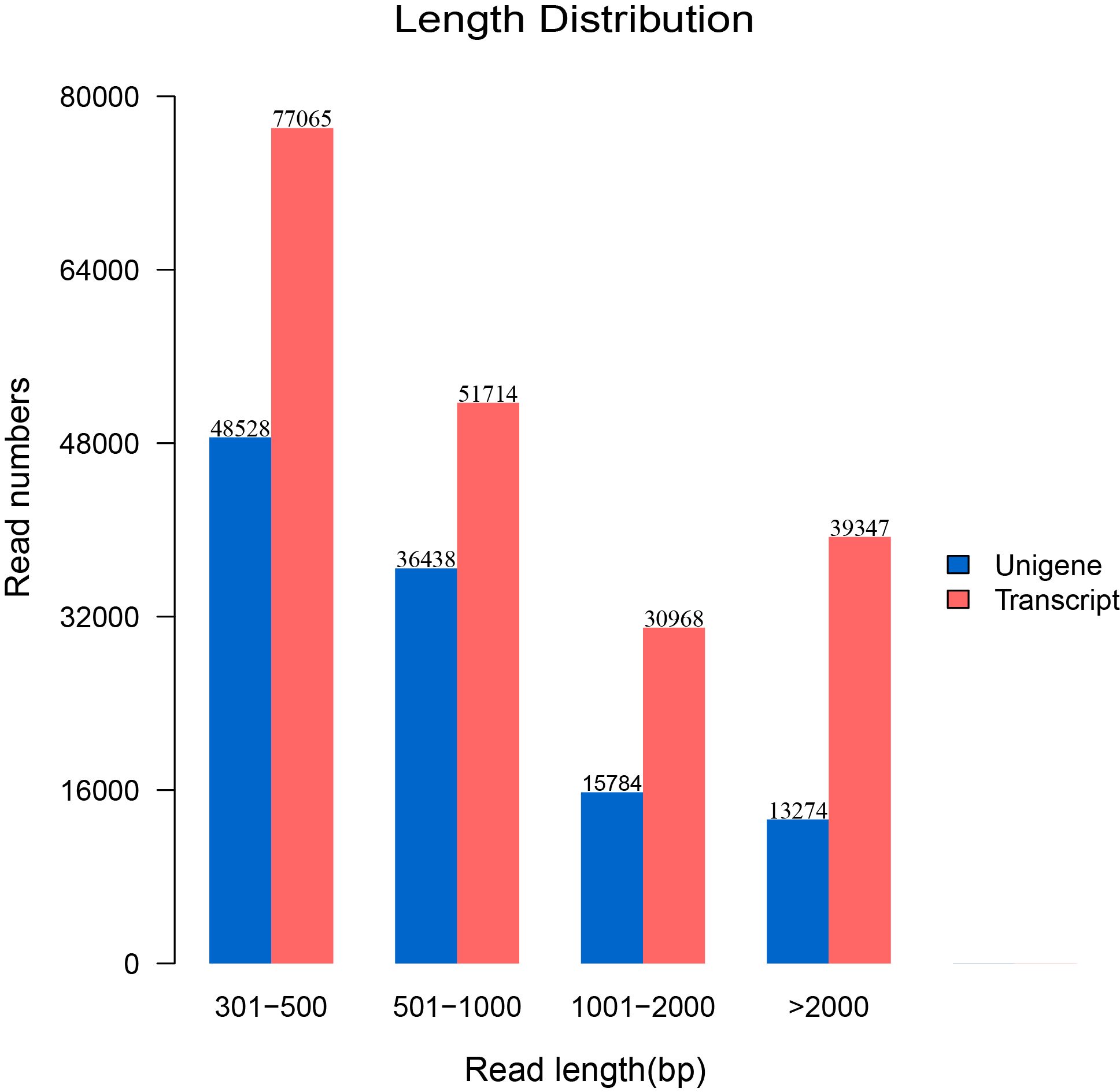


**Supplementary Figure S2.**


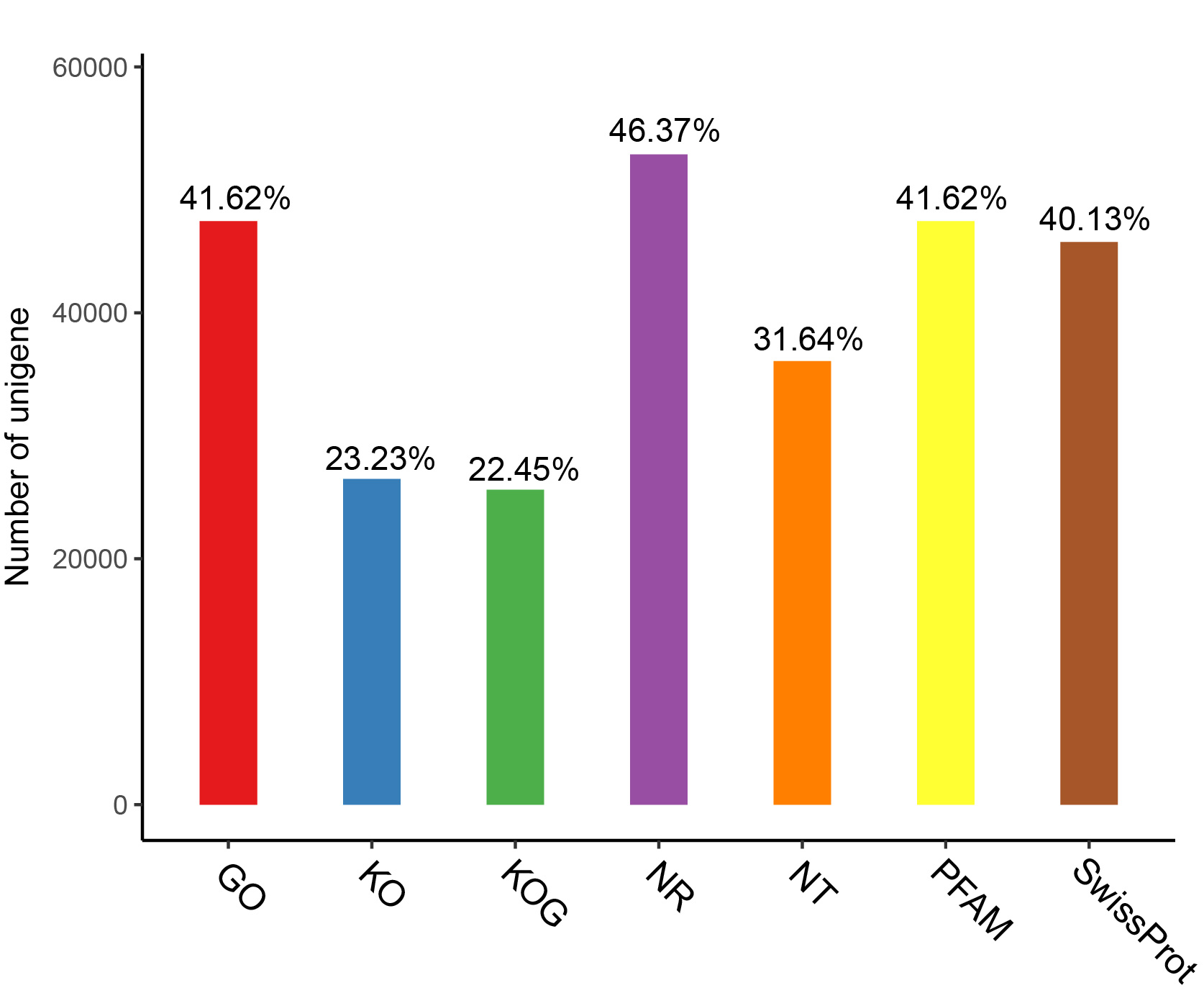


**Supplementary Figure S3.**


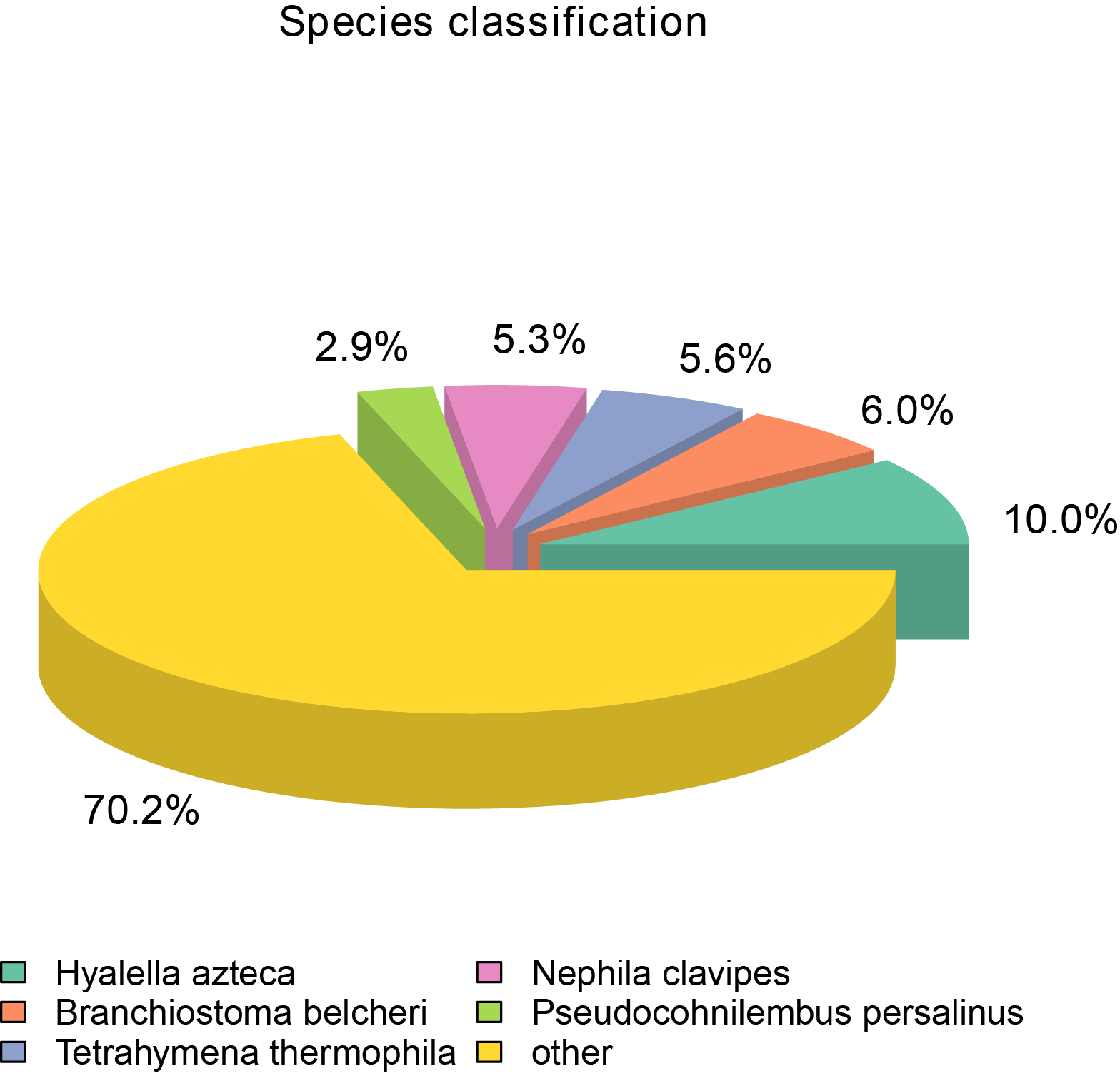

Supplement: Supplementary file 1 — Supplementary Figures. [file 41598_2020_78191_MOESM1_ESM.docx]
